# Bimetallic Organic Gel for Effective Methyl Orange Dye Adsorption

**DOI:** 10.3390/gels10030208

**Published:** 2024-03-19

**Authors:** Hua Jin, Xinyuan Xu, Xiaoyang Yu, Shihua Yu, Shanshan Wang, Xiaoshu Qu

**Affiliations:** Jilin Institute of Chemical Technology, 45 Chengde Street, Jilin 132073, China; xuxinyuan@jlict.edu.cn (X.X.); yangyangyu@jlict.edu.cn (X.Y.); ysh@jlict.edu.cn (S.Y.); wss309778064@126.com (S.W.); xiaoshuqu@jlict.edu.cn (X.Q.)

**Keywords:** organic gel, binary, adsorption, dye removal, water treatment

## Abstract

A bimetallic organic gel (MOG-Fe/Al) was synthesized through the solvothermal method. The gel state of the product obtained under optimized gel formation conditions is sufficient to carry 2 g of weight for a long time. Scanning electron microscopy (SEM), X-ray diffraction (XRD), Fourier transform infrared (FT-IR) spectroscopy, Brunauer–Emmett–Teller (BET) technique, and X-ray photoelectron spectroscopy (XPS) analysis confirmed the structures and morphologies of the synthesized materials. MOG-Fe/Al, with good stability, excellent durability, and wide applicability, exhibited efficient MO adsorption capacity as high as 335.88 mg/g at 25 °C. Adsorption-influencing factors including solution pH, contact time, and temperature were investigated. The adsorption performance of the bimetallic organic gel was better than that of the monometallic organic gels (MOG-Fe and MOG-Al), and its adsorption processes were in accordance with the pseudo-second-order kinetic and Langmuir isothermal models. The excellent adsorption capacity of the MOG-Fe/Al is due to its surface structure, pore volume, π-π interactions, hydrogen bonds, and electrostatic interactions.

## 1. Introduction

In recent years, synthetic dyes have become one of the important pollutants in industrial wastewater from different operations, such as printing, dyeing, textile production, and papermaking [1,2,3]. They usually contain complex aromatic ring structures, and their long-term existence in the environment not only poses potential risks to ecosystems, but also threatens human health through toxic effects, causing a variety of cancers [4,5]. Methyl orange (C_14_H_14_N_3_SO_3_Na), as a typical azobenzene dye, is widely used in agriculture and industry. The accumulation of MO in water bodies will endanger aquatic organisms and seriously threaten the quality of human drinking water [6]. In addition to strict regulation, sewage treatment materials and technologies are important means to reduce water pollution. Many techniques, such as coagulation [7], ion exchange [8], membrane separation [9], adsorption [10,11], advanced oxidation [12,13], and biodegradation [14], have been explored to purify the water. Among them, adsorption is considered as a promising and effective technique for dye removal due to its advantages of simple operation, low cost, high efficiency, and low environmental impact [15,16]. Designing efficient adsorbents is the key to adsorption techniques.

Metal–organic gels (MOGs) are a class of supramolecular organic gels formed by connecting organic ligands and inorganic units through metal coordination, and they are in a semi-solid immobile state at the macroscale [17,18,19,20]. Owing to their low density, porosity, and large surface, their applications in catalysis [21], gas adsorption [22], sensors [23], and water purification [24] have become a research focus in recent decades [25]. In particular, binary and multiple MOGs have abundant active sites and high porosity, and they exhibit excellent performance owing to the synergistic interaction of multiple metals [26,27,28,29]. The bimetallic MOGs benefiting from MIL-100 (Fe and Al) have a large pore size and high pore volume, which can trap larger molecules and are especially suitable for dye removal in acidic and neutral environments. They may be ideal water-amplifier adsorbents and can adsorb more pollutant molecules than monometallic adsorbents. Inspired by this, here, binary metal–organic gel (MOG-Fe/Al) originating from Fe and Al with 1,3,5-benzentricarboxylic acid was produced by the solvothermal technique. The impact factors of gel formation such as reactant ratio, reaction temperature, and solvent type were carefully studied. And the process of MO adsorption on MOG-Fe/Al was assessed by kinetics, isotherms, equilibrium states, and thermodynamic properties. Finally, the durability of MOG-Fe/Al and the adsorption properties on multiple dyes were investigated, and the adsorption mechanism was deduced. 

## 2. Results and Discussion 

### 2.1. Synthesis of MOGs

Generally, in the process of MOG synthesis, the important factors affecting gel formation include the following: reactant ratio, reaction temperature, solvent type, reaction time, pH, surfactants, and inhibitors [30,31]. In this work, six reactant ratios (1:8, 1:5, 1:4, 1:2, 1:1, and 3:2) were investigated using single-factor experiments, and the results are described in Figure S1. As shown in Figure S1A, the reactant ratio of 1:4 is a critical value beyond which the gel state can be formed. Subsequently, the adsorption performances of the four gel-forming materials (1:4, 1:2, 1:1, 3:2) were compared. As shown in Figure S1B, the adsorption properties of the products varied greatly with the reactant ratio, and the products obtained from 1:2 had the highest adsorption properties. In addition, this conclusion was further verified by the fitting results of the relationship between the reactant ratio and the adsorption efficiency (Figure S1C). Furthermore, DMF, EtOH, and DMSO were screened as solvents for forming gels, and the results are shown in Figure S2. Under the same reaction conditions, the apparent morphology of the gel in the DMF system is perfect, and a semi-liquid product can be seen in the EtOH system, while no gel is formed in the DMSO system. Different reaction temperatures (50–130 °C) were explored in the systems with different reactant ratios. As shown in Figure S3, as the proportion of the metal center increases, the gel formation temperature decreases gradually. The system with a 1:2 reactant ratio can form a stable gel at 100 °C. In summary, a 1:2 reactant ratio (metal salt concentration: 0.11 M; coordinating ligand concentration: 0.22 M), DMF solvent, and reaction temperature of 100 °C were chosen as optimized gel formation conditions. The prepared product was further confirmed as a non-flowing semi-solid gel by the falling ball method (Figure S4). A 24 h observation indicated that the weights did not settle, indicating that the gelation degree of MOG-Fe/Al was good enough. When the external conditions are certain, MOG-Fe/Al can maintain the gel state [32,33].

### 2.2. Characteristics of the Samples

**Figure 1:**
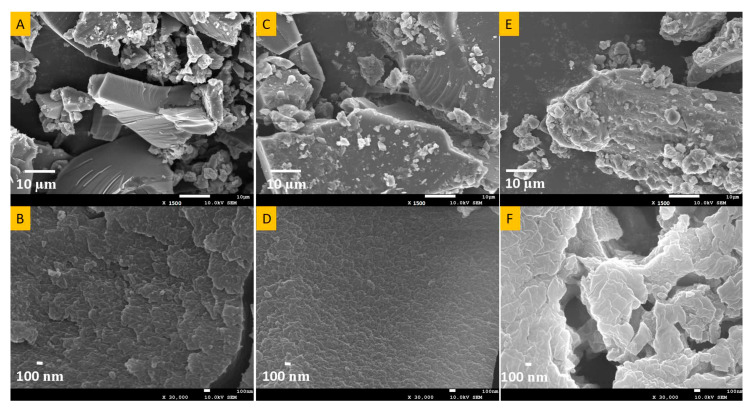
SEM images of MOG-Fe (**A**,**B**), MOG-Al (**C**,**D**), MOG-Fe/Al (**E**,**F**).

The SEM images and EDS spectra of the three materials were assessed, as shown in Figure 1 and Figure S5. The SEM images of monometallic gel materials (Figure 1A–D) indicate that the samples consist of only blocks, which confirmed their phase purity. The blocks exhibit heterogeneous shapes, and their surfaces present dense scale-like textures. By comparison, the surface morphology of bimetallic gel is significantly different; the surface is rough and ragged. Some cavities and channels constructed by the accumulation of blocks provide abundant adsorption sites (Figure 1E,F). In order to confirm the existence of metal components in the gels, the EDS spectra of the three MOGs were obtained. The EDS results show that the three samples contain Fe, C, and O (MOG-Fe); Al, C, and O (MOG-Al); Fe, Al, C, and O (MOG-Fe/Al), respectively, which proved the successful preparation of the organometallic gel (Figure S5). 

**Figure 2:**
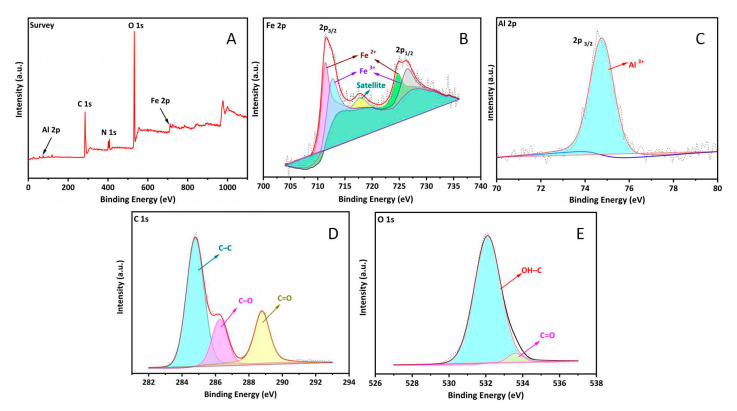
The high-resolution survey (**A**), Fe 2p spectrum (**B**), Al 2p spectrum (**C**), C 1s spectrum (**D**), and O 1s spectrum (**E**) of MOG-Fe/Al.

High-resolution XPS was further used to evaluate the elemental compositions and electronic valence states of the material (MOG-Fe/Al). The full survey spectrum reveals the presence of C, N, O, Fe, and Al (Figure 2A). The high-resolution Fe 2p spectrum (Figure 2B) confirms the presence of Fe^2+^ (711.3 and 724.7 eV) and Fe^3+^ (712.7 and 726.4 eV), with a satellite peak at 717.8 eV. The peaks observed at 74.5 and 74.9 eV in Figure 2C correspond to Al 2p, indicating the presence of Al–O species. The C 1s XPS spectrum is divided into three peaks at approximately 284.8, 286.3, and 288.8 eV, corresponding to C–C, C–O, and O–C=O bonds, respectively (Figure 2D). Additionally, the peaks at binding energies of 532.1 and 533.6 eV in the O 1s spectrum are ascribed to OH–C and C=O groups, respectively (Figure 2E). 

**Figure 3:**
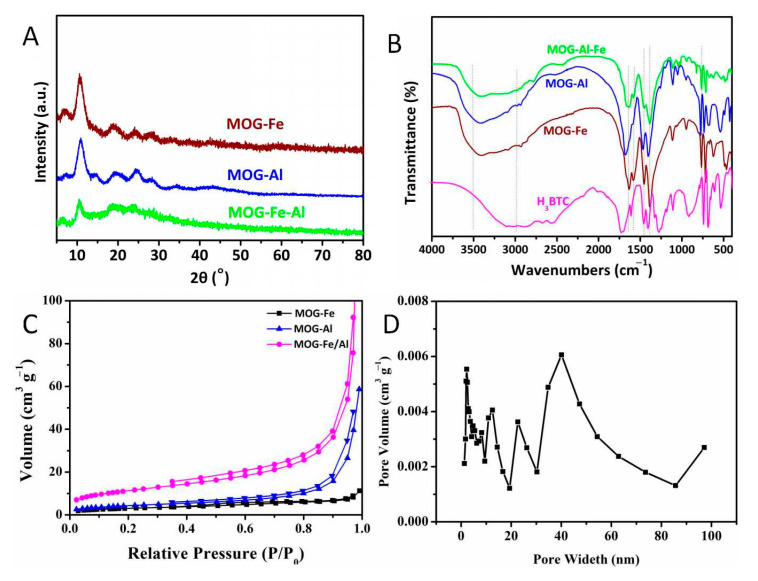
XRD spectra (**A**), FT-IR spectra (**B**), and BET results (**C**) of the three MOGs and pore size distribution of MOG-Fe/Al (**D**).

As shown in Figure 3A, the broad diffraction peaks of MOG-Al, MOG-Fe, and MOG-Fe/Al in the XRD spectra indicate the amorphous nature of the gels, which is consistent with the SEM results. The FT-IR spectra of H_3_BTC and the three MOGs are shown in Figure 3B. By comparison with the IR spectrum of H_3_BTC, a series of characteristic peaks of carboxylate group stretching vibrations can be observed in the three MOGs’ spectra (i.e., a strong peak near 1610 cm^−1^, a doublet in the range of 1440–1370 cm^−1^ and a peak at 770 cm^−1^ coming from the stretching vibrations of the benzene ring in H_3_BTC) [28]. It is noteworthy that the characteristic peak of pure H_3_BTC at 1710 cm^−1^ has shifted to lower wavenumbers (1660 cm^−1^) in the case of the MOGs. The redshift of the band at 1710 cm^−1^ indicates that the carboxylate is coordinated. For the prepared MOGs, the peaks observed between 1610 and 1440 cm^−1^ should be assigned to asymmetric (ν_as_) and symmetric (ν_s_) vibrations of COO^−^, respectively. And the Δν (ν_as_(COO^−^) -ν_s_(COO^−^)) value of 170 cm^−1^ indicates the presence of a bidentate bridge coordination mode in the carboxylic acid group [34]. Additionally, the broadening of the diffraction bands in the range of 3510 to 3000 cm^−1^ is related to the stretching vibrations of O–H emanating from H_2_O [35]. 

The N_2_ adsorption/desorption isotherms of MOG-Fe, MOG-Al, and MOG-Fe/Al exhibit type IV behavior (Figure 3C). The specific surface areas of the three samples are 10.9, 15.8, and 40.9 m^2^/g, respectively. Owing to the hybridization of the two metals, the surface of the bimetallic gel becomes more convex and uneven, resulting in an increase in specific surface area and more adsorption sites. Moreover, the pore size distribution result of MOG-Fe/Al (Figure 3D) shows that the bimetallic gel contains micropores, mesopores, and a few macropores, indicative of a hierarchical porous material. Furthermore, TGA was performed to ascertain the thermal stability of MOG-Fe/Al (Figure S6). MOG-Fe/Al experienced five main steps of weight loss. Zone I describes the initial weight loss occurring below 230.6 °C attributed to water removal from the sample surface. In the range of Zone II to Zone V (230–800 °C), very sharp weight losses were observed due to the decomposition of the organic species and the destruction of the structure.

### 2.3. Adsorption Study of Adsorbents for MO

**Figure 4:**
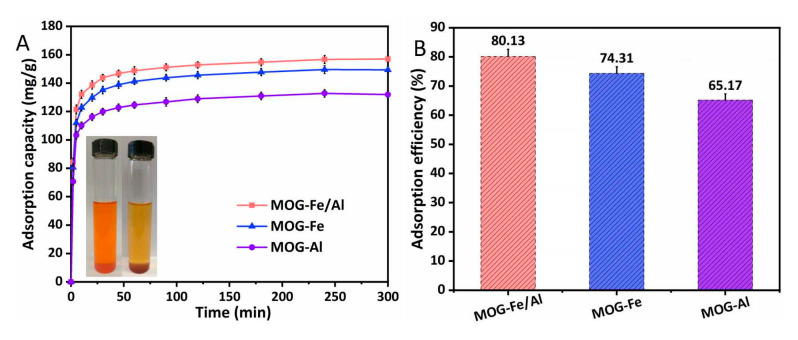
Effect of the metal source on the adsorption properties, Adsorption capacity (**A**) (Inset: picture of the MOG-Fe/Al adsorption system before and after adsorption; *C*_0_: 100 mg/L; adsorbent: 0.5 g/L; pH: deregulation; T: 298 K) and Adsorption efficiency (**B**).

Mono-doped (MOG-Fe and MOG-Al) and dual-doped (MOG-Fe/Al) metal–organic gels derived from Fe and Al as metal sources were successfully synthesized according to the experimental parameters and conditions optimized as described above. As shown in Figure 4A,B, these materials exhibited superior adsorption performance for MO removal. The adsorption capacity of dual-doped gel (156.98 mg/g, adsorption efficiency 80.10%) was higher than that of its mono-doped counterparts (MOG-Fe:148.52 mg/g, adsorption efficiency 74.31%; MOG-Al: 132.8 mg/g, adsorption efficiency 65.17%). Therefore, MOG-Fe/Al was selected as the adsorbent in the subsequent study.

#### 2.3.1. Effect of pH on MO Adsorption

**Figure 5:**
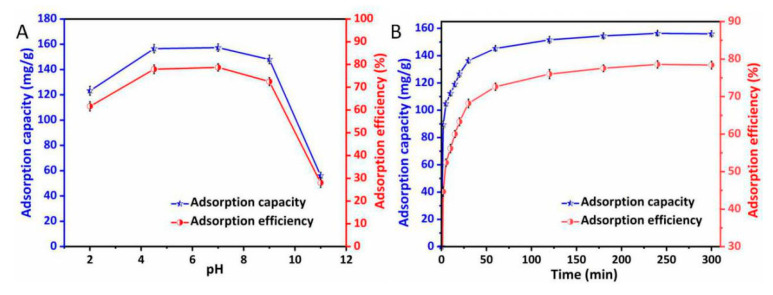
Effect of pH on the adsorption properties (**A**); effect of contact time (**B**) (*C*_0_: 100 mg/L; MOG-Fe/Al: 0.5 g/L; pH: deregulation; T: 298 K).

pH is a crucial factor affecting the adsorption process, which affects the surface properties of the adsorbent and the ionization of the dye [36]. Figure 5A presents the impact of initial pH on MO removal. It reveals that the MO adsorption on MOG-Fe/Al is greatly affected by the variation in pH. It is well known that MO, as an anion dye, exists in the form of sulfonic acid under acidic conditions and in the form of sulfonate under neutral and alkaline conditions. Meanwhile, the surface charge of MOG-Fe/Al at different pH values had been measured, as depicted in Figure S7. The adsorbent was positively charged in the pH range of 2–7. Consequently, electrostatic attraction between the adsorbent and the dye significantly contributed to the adsorption process. Therefore, acidic condition is conducive to MO adsorption, with the adsorption capacity increasing until it reaches 156.02 mg/g. As the pH of the solution further increased to alkaline levels, the surface of the adsorbent became negatively charged, leading to a reduction in MO adsorption due to electrostatic repulsion. Thus, a high MO removal efficiency was maintained within the pH range of 4.5–7 (>78.1%). Considering that the measured pH value of the MO aqueous solution was about 4.5, it is more practical to deregulate the pH value during the adsorption process.

#### 2.3.2. Effect of Contact Time and Adsorption Kinetics 

Contact time is a key factor in determining the efficacy of an adsorbent in actual use and the optimal decolorization time. Batch adsorption experiments were conducted over the range of 5–300 min to investigate the impact of contact time on the adsorption capacity. As shown in Figure 5B, the adsorption capacity of MO increased rapidly in the first 30 min, which is attributed to the abundance of available adsorption sites at first. Subsequently, the absorption process of MO decelerated and reached the equilibrium condition after 240 min, with the highest adsorption capacity of 156.36 mg/g, and then the removal capacity tended to stabilize. Therefore, the optimum contact time for adsorption of MO was 240 min. 

**Figure 6:**
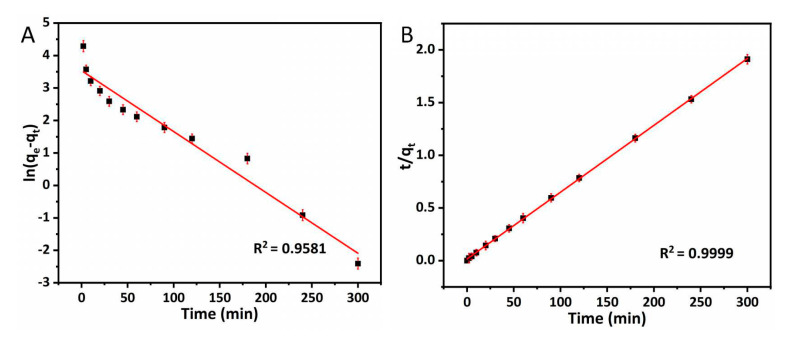
Kinetic fitting curves for MO by MOG-Fe/Al: pseudo-first-order (**A**), pseudo-second-order (**B**) (*C*_0_: 100 mg/L; MOG-Fe/Al: 0.5 g/L; pH: deregulation; T: 298 K).

The kinetic models of MO adsorption on MOG-Fe/Al were compared by the nonlinear regression method, as shown in Figure 6, and the calculated relevant parameters are listed in Table 1. The relatively high *R*^2^ value (>0.99) and the calculated *q_e_* (157.73 mg/g) are basically consistent with the experimental value (156.36 mg/g), indicating that the pseudo-second-order model is more suitable for describing the adsorption phenomenon. It is shown that the chemisorption involving electron exchange or valence forces between the adsorbent surface and the adsorbate is primary in the adsorption process [28].

#### 2.3.3. Effect of Initial Concentration and Adsorption Isotherm 

**Figure 7:**
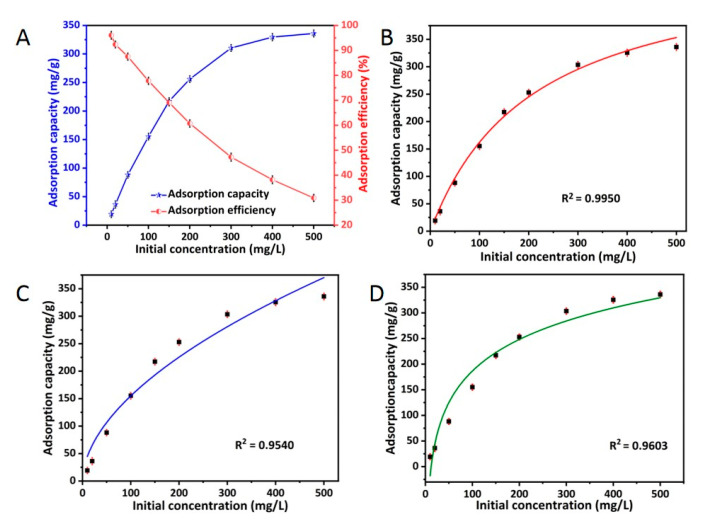
Effect of initial MO concentration on adsorption (**A**) and the Langmuir (**B**), Freundlich (**C**), and Temkin isotherms (**D**) (MOG-Fe/Al: 0.5 g/L; pH: deregulation; T: 298 K).

The impact of the initial MO concentration on the adsorption capacity is shown in Figure 7A. The results showed that with the increase in the initial concentration of dye, the adsorption capacity gradually increased and reached saturation, and the maximum adsorption capacity was 335.88 mg/g. The increment in the initial dye concentration provides mass transfer force and reduces the resistance between the liquid and solid phases. The probability of contact between the dye and the adsorption site is enhanced, which is beneficial for improving the adsorption performance of the adsorbent [37,38]. 

The Langmuir model assumes the possibility of monolayer adsorption, which considers homogeneous adsorption with molecular thickness on the adsorbent. The Freundlich model is often used to discuss multilayer and heterogeneous adsorption processes. The Temkin model is similar to the Freundlich model in that it is suitable for multilayer adsorption except for extremely low and extremely high adsorbent concentrations. In this work, the Langmuir, Freundlich, and Temkin isotherm models were employed to determine the adsorption ability of MOG-Fe/Al (Figure 7B–D). The relative parameters presented in Table 2 indicate that the MO adsorption isotherm fitted the Langmuir model well with the highest *R*^2^ (> 0.99) compared with the Freundlich and Temkin isotherm models (>0.95 and >0.96), and the maximum adsorption capacity was 337.47 mg/g. This result suggests that MO adsorption on MOG-Fe/Al involved monolayer coverage at a specific homogeneous site.

#### 2.3.4. Effect of Adsorption Temperature and Thermodynamics

**Figure 8:**
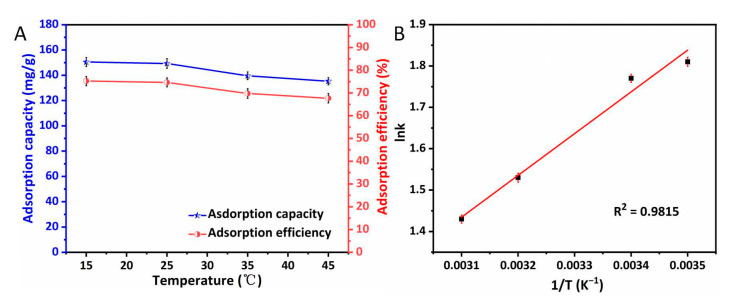
Effect of temperature on MO removal (**A**); Van’t Hoff plot for MO adsorption on MOG-Fe/Al (**B**) (*C*_0_: 100 mg/L; MOG-Fe/Al: 0.5 g/L; pH: deregulation).

The adsorption temperature is also an important factor influencing MO adsorption. The effect of temperature and the thermodynamic data were investigated, as exhibited in Figure 8A. In the temperature range of 15 to 45 °C, the adsorption capacity of MO on MOG-Fe/Al only fluctuated slightly and remained above 150 mg/g. The Δ*S^θ^* and Δ*H^θ^* were acquired from the Van’t Hoff plot (Figure 8B), and the results are presented in Table 3. The Δ*H^θ^* value is negative, indicating that the MO adsorption is an exothermic process. A negative value of Δ*S^θ^* shows that the solid–liquid interaction in the adsorption process reduced the randomness of the system. And negative values of Δ*G^θ^* signify that the adsorption process is spontaneous and feasible. Additionally, as shown in Table 3, the trend of Δ*G^θ^* is positively correlated with the temperature change, indicating that the adsorption process is more favorable at room temperature than at high temperature [39]. Therefore, the adsorption of MO by MOG-Fe/Al is a spontaneous exothermic entropy reduction process.

### 2.4. Durability and Applicability of Adsorbent

**Figure 9:**
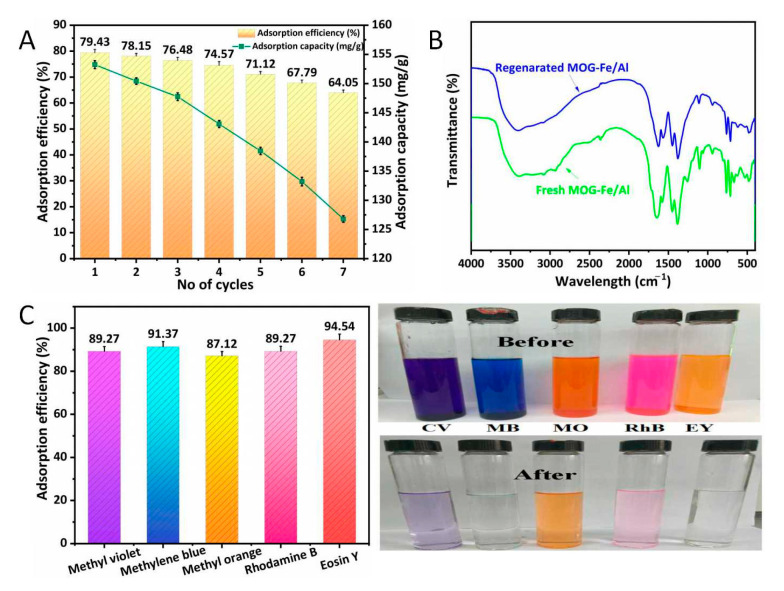
Seven consecutive recycling experiments for MO adsorption by MOG-Fe/Al (**A**); the FT-IR spectra of MOG-Fe/Al before and after regeneration (**B**); adsorption of multiple dyes, i.e., CV, MB, MO, RhB, and EY (*C*_0_ of dye: 20 mg/L; MOG-Fe/Al: 0.5 g/L; pH: deregulation) (**C**).

The durability of adsorbents affects the cost of water treatment operations and is therefore an important factor in evaluating their potential commercial applications. After adsorption of MO, MOG-Fe/Al was removed by centrifugation, regenerated by ethanol-distilled water washing, subjected to separation with centrifugation, and used for continuous recyclable tests. Adsorption–desorption washing was performed in a similar manner for each process. As shown in Figure 9A, the removal efficiency of the regenerated MOG-Fe/Al declined with each cycle and remained above 60% in the seventh cycle. Similarly, the adsorption capacity decreased by 20 percent after consecutive uses. The loss of properties can be attributed to the partial irreversible adsorption of dye on MOG-Fe/Al and incomplete recovery of the adsorption sites. The stability of the adsorbent was explored by detecting the metal leaching of iron and aluminum during the adsorption. The metal loss from the MOG-Fe/Al composite is exhibited in Table S1. As shown in Table S1, the percentage of the leaching element of iron to the total elements was 0.09%, and that of aluminum was 0.37%. Moreover, the FT-IR spectra of the regenerated adsorbent were highly consistent with those of fresh MOG-Fe/Al (Figure 9B). Metal leaching and FT-IR results further confirmed that the adsorbent maintained structural stability during the regeneration process. 

In addition, the adsorption properties of CV, MB, RhB, and EY dyes were also investigated. The concentration changes were measured by UV-Vis, and the characteristic peaks of CV, MB, RhB, and EY appeared at the wavelengths of 584, 665, 554, and 517 nm, respectively. The experimental results showed that the removal efficiency of all dyes was above 87% when the adsorbent dose was 0.5 g/L and the concentration of dye solution was 20 mg/L (Figure 9C). Meanwhile, the adsorption capacity of MOG-Fe/Al was also contrasted with other materials used to remove MO dye from aqueous solutions in previous literature (Table S2). The comparison results showed that MOG-Fe/Al demonstrated wonderful advantages in dosage and adsorption capacity for realizing efficient synthetic dye removal.

### 2.5. Proposed Adsorption Mechanisms

According to the above results of BET, pH, zeta potential, adsorption kinetics, and isotherms, the adsorption of MO on MOG-Fe/Al is mainly chemisorption. FT-IR was conducted to further investigate the interactions between the adsorbent and adsorbate (Figure S8). π-π interaction exists between electron-deficient groups (–SO_3_H in MO and –COOH in MOG-Fe/Al) and electron-rich groups ((CH_3_)_2_NH in MO and –OH in MOG-Fe/Al). After MO adsorption, the characteristic peak of the benzene ring in FT-IR spectra showed a redshift from 1385 to around 1371cm^−1^, indicating the presence of π-π interaction/stacking during adsorption [26]. Hydrogen bonds are typically formed between adsorbents with hydroxyl (–OH), carboxyl (–COOH), and carbonyl (–C=O) groups and contaminants with –OH and –NH groups. After MO adsorption, the FT-IR peak of C=O shifted from 1572 to 1559, and the O–H peak shifted from 3399 to 3395 cm^−1^, indicating the existence of hydrogen bonds [26,27]. Moreover, the pore-filling effect is also very important when using porous adsorbents. MOG-Fe/Al possesses a multi-level pore structure that facilitates the diffusion of dye molecules into the adsorbent and then the capture of these molecules [38]. Furthermore, electrostatic interactions played a role in the adsorption process, and this has been demonstrated by pH influence factor experiments. Overall, the adsorption of MO on MOG-Fe/Al is a complex process, including π-π interactions, hydrogen bonds, pore filling, and electrostatic interactions.

## 3. Conclusions

Monometallic and bimetallic organic gels derived from Fe, Al, and 1,3,5-benzentricarboxylic acid were successfully synthesized using a simple solvothermal approach in this study. The gel state of the product obtained under optimized gel formation conditions (reactant ratio 1:2, solvent DMF, and reaction temperature 100 °C) is strong and stable enough to carry 2 g of weight for a long time. Batch experiments were utilized to investigate the properties of the materials for MO dye removal from water. The results showed that the bimetallic gel (MOG-Fe/Al) exhibited superior adsorption performance to its mono-doped counterparts (MOG-Fe and MOG-Al). The adsorption capacity of MOG-Fe/Al reached 335.88 mg/g under the conditions of 240 min contact time, pH deregulation, dye initial concentration 100 mg/L, adsorbent dose 0.5 g/L, and room temperature. The adsorption process followed pseudo-second-order kinetics (*R*^2^ = 0.9999) and the Langmuir adsorption isotherm (*R*^2^ = 0.9950). Negative values of Δ*H^θ^*, Δ*S^θ^*, and Δ*G^θ^* indicated the spontaneous and exothermic nature of the adsorption process. The excellent adsorption capacity of the MOG-Fe/Al adsorbent is due to the increased surface area and pore volume of the hybrid material, along with the π-π interactions, hydrogen bonds, and electrostatic interactions. Meanwhile, the obtained MOG-Fe/Al adsorbent exhibited good durability and excellent removal for a variety of dyes, such as methylene blue, rhodamine B, eosin Y, and methyl violet. In summary, the bimetallic organic gel MOG-Fe/Al will be an attractive candidate for the removal of synthetic dyes from wastewater in multiple fields.

## 4. Materials and Methods

### 4.1. Materials

Ferric nitrate nonahydrate (Fe(NO_3_)_3_·9H_2_O, ≥99%), aluminum nitrate nonahydrate (Al(NO_3_)_3_·9H_2_O, ≥99%), 1,3,5-benzentricarboxylic acid (H_3_BTC, ≥99%), dimethylformamide (DMF, ≥99%), ethyl alcohol (EtOH), and dimethyl sulfoxide (DMSO) were purchased from Shanghai Titan Scientific Co., Ltd. (Shanghai, China); hydrochloric acid (HCl, ≥99.7%) and sodium hydroxide (NaOH, ≥99%) were obtained from Sinopharm Chemical Reagent Co., Ltd. (Beijing, China); crystal violet (CV, ≥99%), methylene blue (MB, ≥99%), methyl orange (MO, ≥99%), rhodamine B (RhB, ≥99%), and eosin Y (EY, ≥99%) were provided by DaMao Chemical Co., Ltd. (Tianjing, China). All chemicals and solvents were of analytical grade and used as received. Water for experiments was ultrapure water with a resistivity of 18.25 MΩ·cm.

### 4.2. Synthesis of MOGs 

**Scheme 1:**
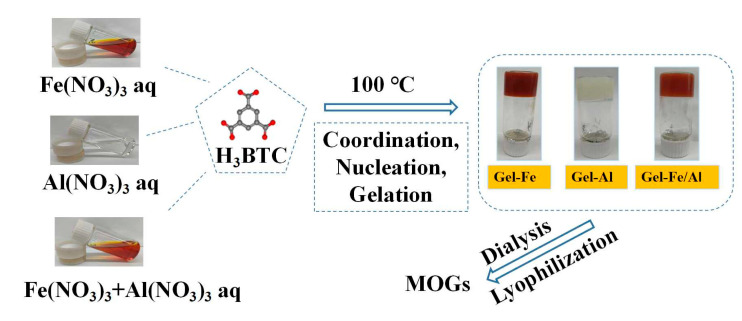
Schematic representation of the procedures for preparing MOGs.

A metal salt mixture (0.75 mmol of Fe(NO_3_)_3_·9H_2_O and 0.75 mmol of Al(NO_3_)_3_·9H_2_O) and 3.0 mmol of H_3_BTC were dissolved in 7 mL of DMF separately with vigorous stirring (600 rpm) at room temperature. The two obtained solutions were then mixed and heated at 100 °C for 6 h and then subjected to natural cooling. The impurities in the initial product were removed by dialysis in ultrapure water, and the xerogel particles obtained after lyophilization were named MOG-Fe/Al. The same procedures were applied for the synthesis of MOG-Al and MOG-Fe, as outlined in Scheme 1. 

### 4.3. Characterization of Adsorbent

The morphology and elemental composition of the samples were studied by JSM-7610F Plus M Field emission scanning electron microscope (FESEM, Tokyo, Japan) coupled with an X-ray energy spectrum analyzer (EDS). Elemental compositions and electronic valence were probed by Thermo Escalb 250 X-ray photoelectron spectroscopy (XPS) with Al Ka radiation at 10 kV and 10 mA. X-ray diffraction (XRD) data were obtained using a Bruker D8 Advance X-ray diffractometer with Cu Kα radiation (*λ* = 1.54 Å) (Karlsruhe, Germany). Thermogravimetric analysis (TGA, Hitachi STA7200; Tokyo, Japan) was performed to measure the thermal stability of gels. Fourier transform infrared (FT-IR) spectra were examined with an Thermo Scientific Nicolet iS50 FT-IR spectrometer (Waltham, MA, USA) in the range of 400–4000 cm^−1^ at room temperature. N_2_ adsorption/desorption isotherms were measured via a USA Micromeritics ASAP 2020 apparatus. The mental concentration of leached elements was measured by an inductively coupled plasma spectrometer (ICP-OES). Zeta potentials were determined on a Malvern Zetasizer Nano ZS90 instrument (Malvern, England). The concentration of MO solution was measured with a UV-Vis UV-2550 spectrophotometer (Tokyo, Japan). The pH of the solution was tested by a Gottingen Sartorius PB 220 pH meter (Munich, Germany). 

### 4.4. Adsorption Experiments

The adsorption characteristics of MO in aqueous solution were assessed through batch adsorption experiments. A given amount of adsorbent (20 mg) was added in an aqueous solution (40 mL, pH 2–11, time periods 5–300 min) containing MO in a concentration range of 10–500 mg/L at a temperature from 15 to 45 °C (288–318 K). After reaching the desired adsorption time, 1.0 mL of the sample solution was extracted, and then the adsorbents were filtered by centrifugation. The MO concentration was determined by using a UV-Vis spectrophotometer at 463 nm. The pH of the solution was adjusted with HCl aqueous solution (0.1 M) or NaOH aqueous solution (0.1 M) at the beginning of the adsorption process. The equilibrium adsorption amount (*q_e_*, mg/g) was calculated using the following equation [29]:

(1) $$q_{e}=\frac{\left(C_{0}-C_{e}\right)}{m}\times V$$

where *C*_0_ and *C_e_* (mg/L) represent the initial and equilibrium dye concentrations, respectively; *m* (g) is the mass of the adsorbent; and *V* (L) is the volume of the dye solution. 

The kinetic studies were performed at 298 K for 5 h. The process was investigated using pseudo-first-order and pseudo-second-order kinetic models [30].

(2) $$\mathrm{pseudo}-\mathrm{first}-\mathrm{order}\;\mathrm{model}:\;ln(q_{e}-q_{t})=lnq_{e}-k_{1}t$$

(3) $$\mathrm{pseudo}-\mathrm{second}-\mathrm{order}\;\mathrm{model}:\frac{t}{q_{t}}=\frac{1}{k_{2}q_{e}^{2}}+\frac{t}{q_{e}}$$

where *q_e_* (mg/g) is adsorption capacity at equilibrium; *q_t_* (mg/g) is MO adsorption at time *t*; *k*_1_ (min^−l^) and *k*_2_ (g/(mg·min)) are the rate constants of pseudo-first-order and pseudo-second-order reactions, respectively.

### 4.5. Adsorption Isotherm

Langmuir, Freundlich, and Temkin models were used to describe the adsorption experimental data. The equilibrium models can be explained as follows [29,31]:

(4) $$\mathrm{Langmuir}\;\mathrm{formula}:\;\frac{C_{e}}{q_{e}}=\frac{1}{K_{l}q_{m}}+\frac{C_{e}}{q_{m}}$$

(5) $$\mathrm{Freundlich}\;\mathrm{formula}:\;lnq_{e}=\frac{1}{n}lnC_{e}+lnK_{f}$$

(6) $$\mathrm{Temkin}\;\mathrm{formula}:\;q_{e}=Bln(K_{t}C_{e})$$

where *C_e_* (mg/L) is the equilibrium concentration; *B* (kJ/mol) is the adsorption heat factor; *q_m_* (mg/g) is the maximum amount adsorbed; *K_l_* (L/mg), *K_f_* ((mg/g)/(mg/L)^1/n^), and *K_t_* (L/g) are Langmuir, Freundlich, and Temkin adsorption constants, respectively; and *n* is a measure of adsorption intensity. 

The thermodynamic evaluation of the experimental results was carried out; that is, the entropy (*S^θ^*), the standard enthalpy (*H^θ^*), and the free energy (*G^θ^*) were obtained using the following equations:

$$\Delta G^{\theta }=-{RTlnK}_{l}$$

$$\Delta G^{\theta }=\Delta H^{\theta }-T\Delta S^{\theta }$$

$$lnK=\frac{\Delta S^{\theta }}{R}-\frac{\Delta H^{\theta }}{RT}$$

## Figures and Tables

**Table 1:** Kinetic parameters for MO adsorption by MOG-Fe/Al.

Pseudo-First-Order Model			Pseudo-Second-Order Model		
*k*_1_ (min^−1^)	*q_e,_*_1_ (mg/g)	*R* ^2^	*k*_2_ (g/mg·min)	*q_e,_*_2_ (mg/g)	*R* ^2^
–1.94 × 10^−2^	46.63	0.9581	8.5 × 10^−4^	157.73	0.9999

**Table 2:** Parameters of Langmuir, Freundlich, and Temkin isotherms.

Langmuir			Freundlich			Temkin		
*K_l_* (L/mg)	*q_m_* (mg/g)	*R_l_* ^2^	*K_f_* (mg/g)/(mg/L)^1/n^)	*n*	*R_f_* ^2^	*K_t_* (L/g)	*B* (kJ/mol)	*R_t_* ^2^
0.00576	337.47	0.9950	13.80	1.92	0.9540	0.081	88.84	0.960

**Table 3:** Thermodynamic parameters for MO adsorption by MOG-Fe/Al.

T/(K)	Δ*G^θ^*/(kJ/mol)	Δ*H^θ^*/(kJ/mol)	Δ*S^θ^*/(kJ/mol)
288	−4.39	−8.39	−1.69
298	−4.34		
308	−3.92		
318	−3.78		

## Data Availability

The original contributions presented in the study are included in the article, further inquiries can be directed to the corresponding authors.

## Supplementary Materials

The following supporting information can be downloaded at https://www.mdpi.com/article/10.3390/gels10030208/s1, Figure S1: Selection of reactants ratio; Figure S2: The result of solvents screening; Figure S3: The result of Tgel screening; Figure S4: The result of falling ball experiment of MOG-Fe/Al; Figure S5: EDS spectra of MOG-Fe (A), MOG-Al (B), MOG-Fe/Al (C); Figure S6: The TGA curve of MOG-Fe/Al; Figure S7: Zeta potential at different pH; Figure S8: The FT-IR spectra of MOG-Fe/Al before and after MO adsorption; Table S1: Homogenous processes by leached iron and aluminum from the MOG-Fe/Al; Table S2: Comparison of MO adsorption capacities of with MOG-Fe/Al other reported adsorbents [40,41,42].
