# Supplementary material for: Bimetallic Organic Gel for Effective Methyl Orange Dye Adsorption
Source: Gels. 2024 Mar 19;10(3):208. doi: 10.3390/gels10030208 (PMC10970059; doi:10.3390/gels10030208)
Supplement: Supplementary file 1 [file gels-10-00208-s001.zip › gels-2879536-supplementary.pdf]

Supplementary Information: Bimetallic Organic Gel for Effective Methyl orange Dye Adsorption

Hua Jin*, **Xin-Yuan Xu, Xiao-Yang Yu, Shi-Hua Yu, Shan-Shan Wang, Xiao-Shu Qu**

Jilin Institute of Chemical Technology, 45 Chengde Street, Jilin, 132073, China

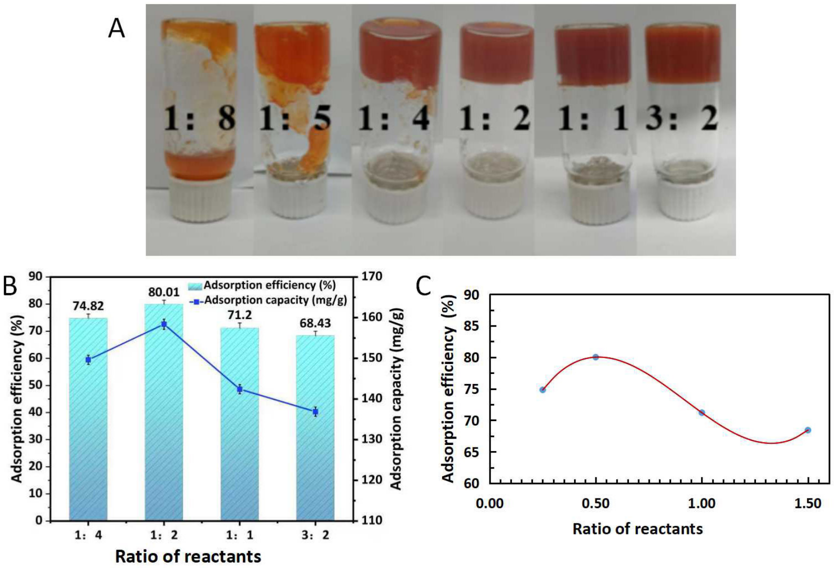

Figure S1. Products synthesized under different reactants ratiot (under the same synthesis conditions) (A); Adsorption efficiency of the products (reactant ratios of 1:4, 1:2, 1:1, and 3:2, respectively) (B); Fitting results of the relationship between the reactant ratio and the adsorption efficiency (C) (C_0 (MO): 100 mg/L; adsorbent: 0.5 g/L; pH: deregulation).

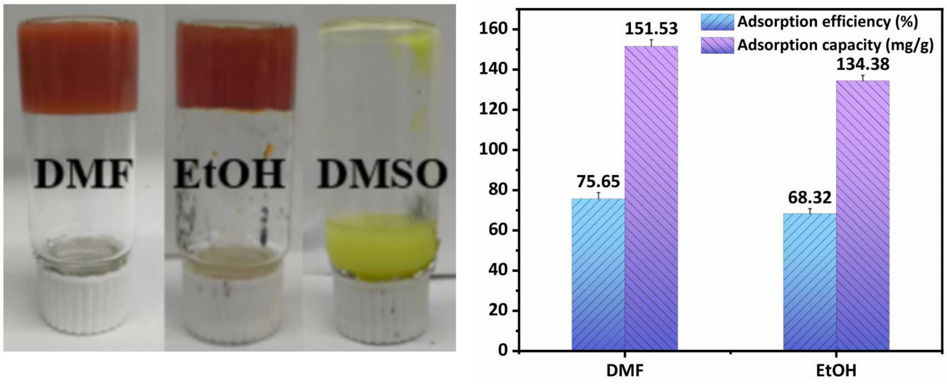

(A) **(B)**

Figure S2. Products synthesized in different solvents (under the same synthesis conditions) (A); Adsorption performance of products from DMF and EtOH (B) (C_0: 100 mg/L; adsorbent: 0.5 g/L; pH: deregulation).

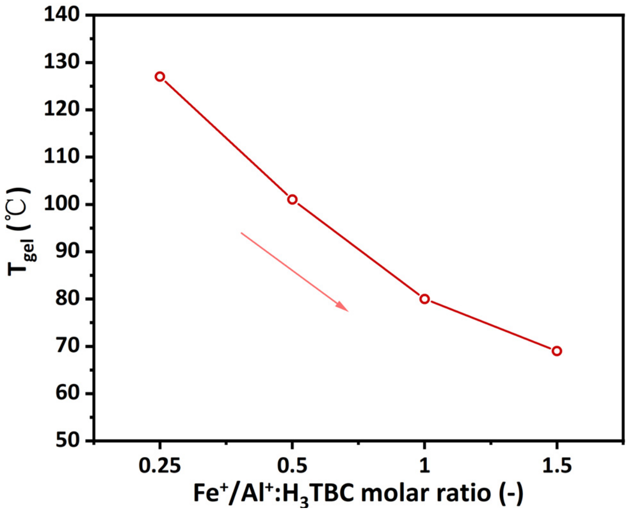

Figure S3. The result of Tgel screening (MOG-Fe/Al, reactant ratios:1:4, 1:2, 1:1, and 3:2).

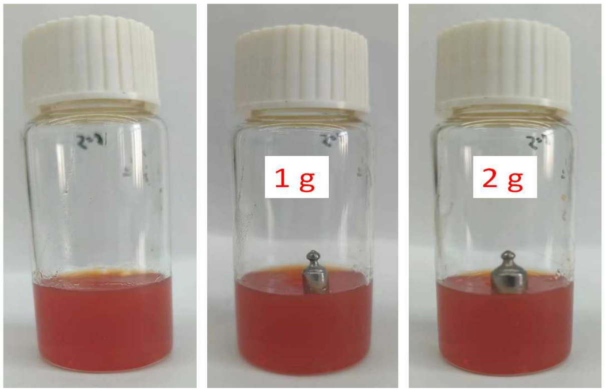

Figure S4. The result of falling ball experiment of MOG-Fe/Al (Observation time: 24 h, 1 g and 2 g weights were used).

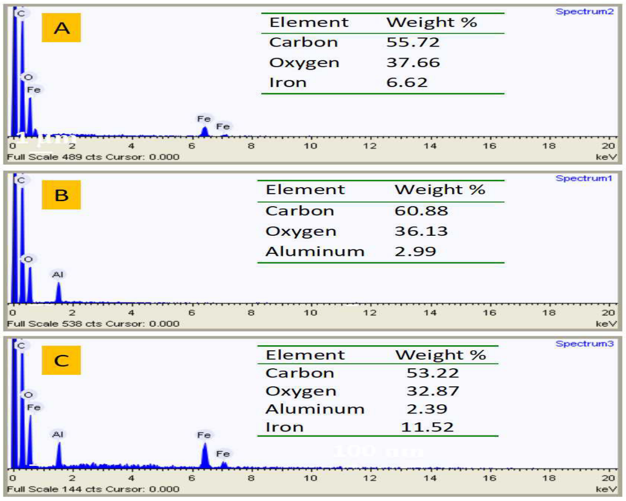

Figure S5. EDS spectra of MOG-Fe (A), MOG-Al (B), MOG-Fe/Al (C).

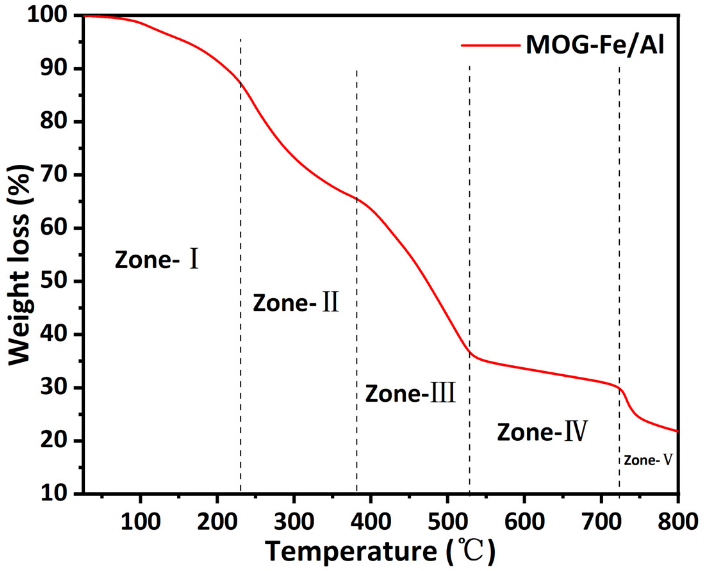

Figure S6. The TGA curve of MOG-Fe/Al.

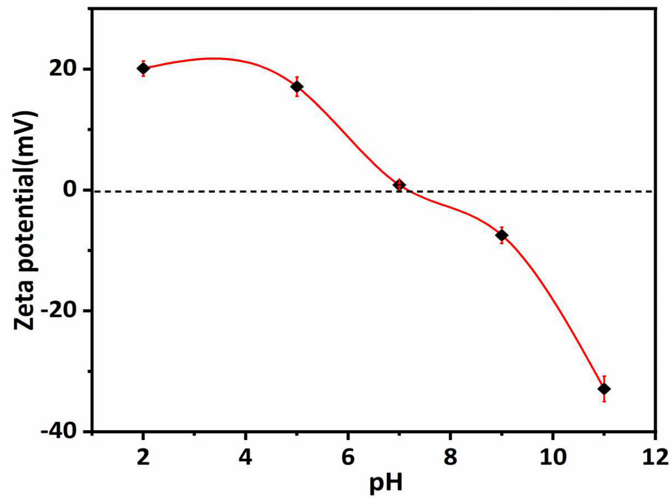

Figure S7. Zeta potential at different pH (adsorbent: 0.5 g/L; pH: deregulation; T: 298 K).

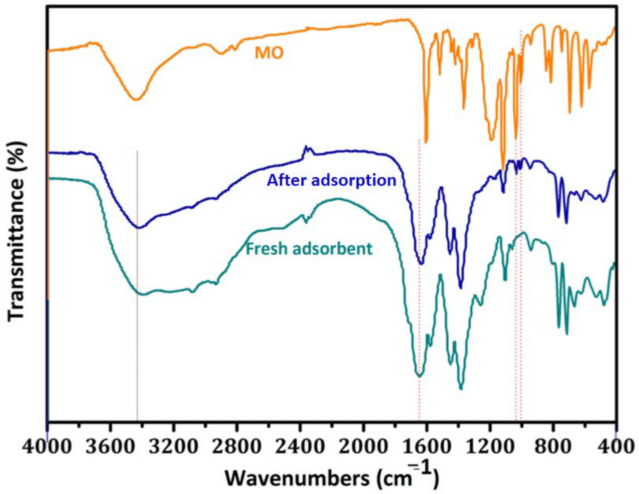

Figure S8. The FT-IR spectra of MOG-Fe/Al before and after MO adsorption.

Table S1 – Homogenous processes by leached iron and aluminum from the MOG-Fe/Al.

**Metal element**	**Fe**	**Al**
**Mass of fraction of element[a] (%)**	11.52	2.39
**Concentration of leached element[b] (mg/L)**	0.0512	0.0449
**Leached element percentage of total element[c] (%)**	0.09	0.37

a: Mass fraction of element: tested by EDS.

b:Concentration of leached element: tested by ICP-OES.

c:Leached element percentage of total element: calculated with 20 mg adsorbents and by the equation percentage = $\frac{m_L}{m_T} \times 100\ \%$

m_L= leached element content (mg), m_T = total element content in the fresh adsorbents (mg).

Table S2 – Comparison of MO adsorption capacities of with MOG-Fe/Al other reported adsorbents.

Adsorbent	Dosage (g/L)	Pollutant concentration (mg/L)	Adsorption capacity (mg/g)	References

Zn -MOG	2	50	100	Colloid surface A. **2021**, *628*, 127335 [40]
Bimetallic MOG	1	50-200	265	Sci. Rep. **2015**, *5*, 10556. [24]
Magnetic porous carbon (MPC)	0.5	12.5-400	182.82	Dalton Transactions, **2016**, *45*, 4541-4547 [41].
Three-dimensional graphene aerogel (TCGA)	1	100	56.4	New Journal of Chemistry, **2020**, *44*, 16285-16293. [42]
MOG-Fe/Al	0.5	10-500	335.88	This work
